# 

**Published:** 1899-12

**Authors:** 


### MARRIAGES.

At Philadelphia, November 29, 1899, by the Rev. J. Joyce Moore, Dr. Francis S. Allen and Miss Mabeth Evangeline Todd.

On November 29, 1899, I. C. Newhard, V. S., of Ashland, Pa., and Miss Jennie E. Price, of Harrisburg, Pa.

### COMMUNICATIONS.

### PERSONALS.

Dr. F. H. Schneider, of Ashland, Pa., is now a resident and practitioner in Philadelphia, having recently removed to this city.

Dr. G. W. Browning, of Vandalia, Ill., has removed to Rome, Ga., where he will practice in the future.

Dr. A. B. Kelly, formerly of Cohoes, N. Y., has for the past year been assisting his brother, Dr. Wm. H. Kelly, of Albany, N. Y.

Dr. H. D. Martein, of Philadelphia, was severely injured in the face while attending one of his equine patients in November.

Dr. W. B. Kille, of Woodstown, N. J., succeeds to the practice of Dr. R. G. Webster at Salem, in the same State.

Dr. J. C. McNeil, of Pittsburg, Pa., in the capacity of Examiner for the State Livestock Sanitary Board, has contributed much in brushing away obscure and uncertain points as to the use of tuberculin under many conditions where the accuracy as a diagnostic agent has heretofore been in doubt.

Drs. R. S. Huidekoper, H. D. Gill, and T. Delaney, of New York, figured as the experts in a recent suit in Brooklyn as to the warranty and sale of a show-ring horse.

Dr. Jos. N. Megary, of Baltimore, has become associated with the practice of Dr. George N. Cornelius, of the Oriole City.

Dr. Alex. Glass, of Philadelphia, enjoyed a week's sport in November in the field, following the ofttimes uncertain luck with the gun.

Drs. D. S. White, W. F. Lavery, G. B. Frederick, and G. V. Brumley represent the clinical staff of the Veterinary Hospital of the College of Veterinary Medicine of the Ohio State University.

Dr. B. J. Orr, of Bridgetown, succeeds Jos. Orr, at Baden, Ontario.

Dr. L. A. Klein, recently of Fort Worth, Texas, will succeed Dr. Johnson at the Philadelphia abattoirs as assistant to Dr. Chas. Schaufler, chief inspector.

Dr. D. R. Hoffman, of Baltimore, Md., having embarked in the ice-manufacturing business, disposed of his practice to Dr. Hegerty, of the same city.

Dr. A. F. Schrieber, of Philadelphia, conducted a special test of the famous trained horse "Jim Key" as to his abilities in arithmetical problems early in December.

Dr. Arthur L. Parker, formerly of Providence, R. I., is now located at Riverdale, N. H.

Dr. S. L. Blount, formerly of Texas, has recently located at Martinsburg, W. Va.

Drs. W. W. Thorburn, of Lansing, John F. Deadman, of Sault Ste. Marie, and George Hare, of Allegan, have been appointed as the Board of Veterinary Medical Examiners of Michigan by Governor Pingree.

Dr. J. Stewart Lacock, of Allegheny City, Pa., was among the exhibitors at the Philadelphia dog-show in November.

Dr. Harry Lukes, of Springfield, Mass., was recently summoned across the Atlantic, incidental to the illness and subsequent death of his father.

Dr. J. W. Harrigan, of Philadelphia, who is now the proprietor of some four drug stores in the "Quaker City," finds relief from the exactions of his business behind a fast trotter, and occasionally tests his metal on the track with other fast pace-makers.

Dr. Leonard Pearson, of Philadelphia, and M. E. Conard, of West Grove, Pa., addressed the convention of State Agriculturists of West Chester, Pa., in November.

Dr. R. L. Tritton, of Richmond, Va., is the owner and admirer of a steeple-chaser, and frequently tries his prowess with others of the same class. He officiated as judge of the jumpers at the Deep Run Hunt Club in November.

Dr. George Hare, of Allegan, Mich., recently appointed a member of the Michigan State Board of Veterinary Medical Examiners, is a graduate of the Grand Rapids Veterinary College.

Drs. R. S. Huidekoper, E. M. Ranck, Leonard Pearson, C. J. Marshall, M. Jacobs, Wm. J. Denton, and M. E. Conard were among the Thursday evening visitors of the Philadelphia dog-show.

Dr. H. Clay Glover, of New York City, is a frequent visitor to the speedway, and has a very fast-going gray gelding, with which he delights to meet his friends.

Dr. James Johnson, recently located at the port of Philadelphia, inspecting meat for foreign export, has been transferred to Boston.

Dr. C. J. Marshall, of Philadelphia, was the official veterinarian to the dog-show at that point in November.

Dr. H. B. Shugar, of Lebanon, Pa., was a visitor to Philadelphia in the latter part of November.

Dr. Fred. L. Felber, of Baltimore, is a frequent visitor to Philadelphia, and dame rumor has it that the fair sex of the "Quaker City" have charms for him. Dr. Felber is the coming city veterinarian of Baltimore.

Dr. John J. Repp, of Ames, Ia., addressed the Iowa Improved Stock Breeders' Association in December on "Ounces of Prevention in Caring for Domestic Animals."

Drs. John J. Maher, Jas. G. Fairley and a large delegation of veterinary students were attendants at the dog-show in Philadelphia in November.

Dr. H. A. Meisner, of Baltimore, was a recent visitor to Philadelphia's noted horse-markets, selecting coach-horses for his clientèle.

Dr. Leonard Pearson addressed the Farmers' Institute at Cannonsburg, Pa., on the subject of "Bovine Tuberculosis, and How it may be Eradicated from Dairy Herds."

Dr. R. W. Carter, of Jobstown, N. J., is the owner of several fast runners, and finds profit and pleasure in following their achievements in the stud and on the track.

Veterinarian Chandler Quintin, of Newport, R. I., is the owner and directs the affairs of the Newport Riding Academy.

Among those who passed the examination for first-class veterinarian under the recent army requirements were the late Dr. M. J. Treacy, Dr. Wm. V. Lusk, Dr. Gerald E. Griffin, Dr. C. D. McMurdo, and Dr. D. Lemay. Among those passing a second-class veterinarian's examination were Drs. Wm. J. Waugh and Foster.

Dr. Henry Bower, of Philadelphia, has become "mine host" of an extensive hotel plant at Collegeville, Pa., where he is prepared to provide for the wants of man and beast at all hours.

### INDEX.

ABSCESS from foreign body in the dog, 362
(needle) in the horse, 362
Absorption and excretion of iron, 432
Acetic acid, fluid extracts made with, 655
Ackerman, Edwin B., D.V.S., 75, 402, 742
Acoin, 365
Action of boric acid and borax on pepsin and pancreatin, 434
Actinomycosis, 758
Active membership in a veterinary association, advantages of, 71
Acute indigestion in horses, a new method of employing charcoal in the treatment of, 16
Adulteration of creosote, 166, 369
Advantages of active membership in a veterinary association, 71
Advertise in the Northwest, how they, 527
Agricultural districts, veterinary lectures in, 226
Albumin in urine, simple test for the detection of, 429
Alcohol as an antidote for carbolic-acid poisoning, 366
Alger, Russell A. (signed), 133
Alumni Association, silver anniversary of the A. V. C., 522
Alum powder, compound (Squibb's), prescription for, 106
American Veterinary College, 452
silver anniversary, 370, 457, 522
State of Massachusetts, New England Association of, 398
Veterinary Medical Association, 60, 384, 455, 801
in New York, 313
thirty-sixth annual meeting, 518
Veterinary Medicine, 634
Among the Board of Examiners, 524
the colleges, 382, 523, 593, 733
Amputation of the tail, 103
Anæsthesia in veterinary surgery, a plea for the more general use of, 681
Anæsthetic mixture, E.C.P., 105
Analysis, urinary, 217
Animals, treatise on surgical therapeutics of domestic, 501
Another veterinary journal, 591
Annual dinner of the Westchester County Veterinary Medical Association, 528
Annual Report of the State Veterinarian of Pennsylvania (new publications), 304
Anodyne liniment, prescription for, 105
Antidote for carbolic-acid poisoning, alcohol as an, 366
to carbolic-acid poisoning, camphor as an, 586
Antiseptic, formalin as an, 710
solution, prescription for, 106
Antiseptics, 710
Antisepsis in veterinary practice, practicability of, 482
Antitoxin, tetanus toxin and, 639
Apoplexy, eserine and barium chloride in parturient, 445
nux vomica in parturient, 449
Appointment, an excellent, 46
Arecoline in laminitis, 104
Argentum colloidale Credé, on the treatment of purpura hemorrhagica in horse with, 289
Army bill, the, 170
horses, the course and diagnosis of moon-blindness in, 226
I approve of the organization of a veterinary contingent to, 133
legislation, report of committee on, 598
notes, 303
veterinary service, 537
Arrangement for municipal ownership of slaughter-houses, an, 199
Arsenic, magnesium hydrate as an antidote in poisoning by, 434
Aseptic surgery, advantage of, 590
Ass, an attempt to affect the, with tuberculin, 226
Association of Veterinary Faculties and Examining Boards of N. A., 521
more about, 795
meetings in January, 48
Attempt to affect the ass with tuberculin, 226
Austin, W. H., V.S., 488
Automobile, passing of the, 792
Azoturia, 277, 552
in a dog, case of, 376

BACILLUS, the rabietis, 230
Barium chloride, 87
in parturient apoplexy, eserine and, 445
Beasts, have mercy on dumb, 414
Belladonna, deadly nightshade or, 491
Berlin Tuberculosis Congress, the, 239
Best hen, the, 260
Biology of pathogenic microörganisms, 612
Bitch, ovariotomy on the, 85
Bittles, E E., V.S., 492
Black, Judson, V.S., 445
Blackman, G. B., D.V.S., 155
Board of Examiners, among the, 524
Body, the influence of the mind on the diseases of the, or the difference between veterinary and medical science, 205
"Boiled" linseed oil, 300
Boric acid and borax on pepsin and pancreatin, action of, 434
Bostrom, A., D.V.S., 336
Bovine rumenotomy, 223
tuberculosis, 658
on the relations which exist between human tuberculosis and, 1
Bowels, a case of paralysis of the, 102
Boxes, to fasten labels on tin, 37
Brimhall, S. D., V.M.D., 150
British Medical Journal, epitome of, 303
Brown, John E., 180
Bureau men, work among, 733
Burns, solution for, 364
Butterfield, J. F., V.S., 286
Byers, M. V., V.S., 219

CALCULUS, cystotomy for cystic, 360
Calf, case of fœtal tuberculosis in a, 163
California State Veterinary Medical Association, 51
Calomel, origin of, 716
Camphor as an antidote to carbolic acid, 586
Canadian chicken cholera, 605
Cancer, treatment of, 105
Canine influenza, 774
Carbolic acid in the treatment of tetanus, 235
poisoning, alcohol as an antidote to, 366

Carbolic acid poisoning, camphor as an antidote to, 586
treatment of, 436
Carbo-sapol, 367
Career, the end of a noble, 373
Case-book and reporting cases, on the importance of keeping, 547
Casper, Dr. A. M., 464
Castrating horses, a new method of, 589
Castration of cryptorchids (ridglings), 286
for prostatic hypertrophy, 233
Cat, "distemper" in the, 361
Cathartics, hypodermic, 26, 87
Catheterization, 783
Cattle, a few notes on foreign bodies swallowed by, 407
dairy, tuberculosis in, and how shall we get rid of it, 413
rabies in, 787
to protect from flies, 586
Cellulitis among horses, epizoötic, 511
Cerate of cantharides, 585
Charcoal in the treatment of acute indigestion in horses, a new method of employing, 16
Chicago Veterinary Society, 52, 180, 390, 463, 530, 594, 731
Chinosol, 301
Chloride of iron, tincture of, 585
Chloroform, decomposition of, 583
testing the purity of, 713
water, cocaine hydrochlorate incompatible with, 365
Choking, case of, 448
Chronic hydrocephalus in a horse, 38
Clancy, Dr. Joseph B., 186, 391, 464, 531, 594, 732
Clark, W. G., M.D.C., 316
Clayton, C. E., D.V.S., 124, 273
Clinical diagnosis of nasal discharges, 441, 507
of stringhalt, 112
of tumors, 210
on the importance of, 36
lectures, 686
Clinical reports:
Calculus, cystotomy for cystic, 360
Cat, "distemper" in the, 361
Colon, impaction from adhesion of the small, 363
Dog, abscess from foreign body in the, 623
Eyeball, excision of the, 233
Harger, S. J. J., V.M.D., 231, 360
Horse, "needle" abscess in the, 362
sciatic neurectomy in the, 231
Hypertrophy, castration for prostatic, 233
McNeall, J. H., V.M.D., 231
Pennsylvania Veterinary Hospital, University of, 231, 360
Shoe-boil, surgical treatment of, 232
College and society notes, 48
Colleges, among the, 382, 593
Colon, impaction from adhesion of the small, 363
Coming events, 455
Communications, 239, 450, 592, 709, 793
Commencement exercises:
Grand Rapids Veterinary College, 310
Kansas City Veterinary College, 523
Indiana Veterinary College, 305
McKillip Veterinary College, 306
New York College of Veterinary Surgeons, 305
Ontario Veterinary College, 311
United States College of Veterinary Surgeons, 306
University of Pennsylvania, Veterinary Department, 458
Veterinary Department of McGill University, 307
Complete eversion of uterus in a mare, 378
Compound alum powder (Squibb's), prescription for, 106
tincture of opium, prescription for, 585
Cocaine hydrochlorate incompatible with chloroform water, new test for cocaine, 365
solution, 369
Concentrated feed-stuffs (new publications), 122
Congress, the Berlin, 239
Conjunctivitis, solution for, 106
purulent, 106
Constituents of digitalis leaves, 582
Consultation, ethics of, 747
Contribution to the study of the infectiousness of milk from tuberculous cows and as to the value of the tuberculin test, 572
Cork-stoppered bottles, 584
Corn-stalk diseases, 336
Corrections, 729
Correspondence, 42, 115, 381
Corrigan, John, 85
Cotton-seed disease, 560
Counter-irritants, 751
Cough, prescription for, 169
Creoline as a deodorant of iodoform, 433
Creosote, adulteration of, 166, 369
pills, prescription for, 106
soluble in water, to render, 433
Cribbing and its operative treatment, 22
myoneurectomy for, 720
Crotalism, 592
Cryptorchids, castration (ridglings), 286
Cystotomy for cystic calculus, 360

Dairy cattle, tuberculosis in, and how shall we get rid of it? 413
sanitation, 473
Dalrymple, W. H., M.R.C.V.S., 265, 350, 625
Day, C. M., D.V.M., 473
Deadly headache powders, 586
nightshade, or belladonna, 491
Decomposition of chloroform, 583
Deodorized petroleum oil, 365
Department of Surgery
Antiseptics, 107
Catheterization, 783
Clinical diagnosis of nasal discharge, 441, 507
on the importance of, 36
Jay, Robert, M.D.V., 437, 507, 587, 717, 783
Merillat, L. A., V.S., 36, 37, 107, 112, 297, 437, 441, 507, 587, 717, 783
Quittor, 717
Sloan, J. A., B.S., M.D.V., 437, 507, 587, 717, 720, 783
Stringhalt, clinical diagnosis of, 112
Surgical items, 114, 299, 444, 509, 589, 720, 786
Trephining, its indications, technique, after-treatment, and sequelæ, 297, 437, 587
Wyman, W. E. A., M.D.V., V.S., 36, 107, 114, 297, 299, 437, 444, 507, 587, 717, 783
Dermatitis gangrenosa, or gangrenous grease, 469
Desmond, Veterinary Surgeon, 497
Destruction of jugular vein and carotid artery following the intravenous injection of barium chloride, 512
Detection of albumin in urine, a simple test for, 429
Diagnosis of nasal discharges, clinical, 441, 507
of tuberculosis, for a more accurate, 434
on the importance of clinical, 36
Dickson, Robert, D.V.S., 376
Dieckerhoff, Dr. W., 289
Dietetics, 625, 699
Digitalis, 67
leaves, constituents of, 582
Discharges, clinical diagnosis of nasal, 441, 507
Disease, cotton-seed, 560
of rabbits, parasitic, 497
Diseases, corn-stalk, 336

Diseases of animal acts, 780
of the udder and teats, 488
Disinfection, 674
"Distemper" in the cat, 361
District of Columbia, Veterinary Association of the, 129, 324
Docking for fashion, shall the veterinarian perform the operation of? 43
Dog, abscess from foreign body in the, 362
case of azoturia in a, 376
operation for constipation on the intestines and stomach of a, 162
soap, prescription for, 584
Doses in the U. S. Pharmacopœia, 713
Drasky, J. J., 282.
Drugs, adulteration of, 712
what becomes of some spoiled, 235
where some come from, 711
which should not be dispensed in capsule form, 583
Dubia, Dr. O. R., 531
Dumb beasts, have mercy on, 414

Echinococcus veterinorum, 215
Eczema, solution for, 586
Eddy, William, 787
Editorials, 46, 118, 119, 171, 238, 371, 452, 454, 791
American Veterinary College, 452
Veterinary College silver anniversary, 370, 516
Another veterinary college, 591
Army bill, the, 170
Appointment, an excellent, 46
End of a noble career, the, 373
Ethical education, 371
Fearful responsibility, a, 513
Health, veterinary legislation and State boards of, 237
Illinois, veterinary legislation in, 372
Michigan's veterinary act, 304
New York in September, 515
in 1899, Greater, 45
New York's triumph. Greater, 657
Nineteen hundred, 791
Now for army recognition, 45
Now for New York, September, 1899, 370
Passing of the automobile, the, 792
Pennsylvania, 453
Profession loss, our, 514
Scotch these bills in New York, 119
"Senate," now for the, 118
Where in 1900? 723
Education, a campaign of, 145
Emmert, J. M., M.D., 145
End of a noble career, the, 373
Electricity and its use in veterinary practice, 419
Ellis, Robert W., D.V.S., 60, 196, 251, 324, 398
Epizoötic cellulitis among horses, 511
Equine medicine, practice of (new publications), 121
Errors in X-ray photographs, 432
Escharotic paste, 300
Eserine, 492
and barium chloride in parturient apoplexy, 445
Ethical education, 371
Ethics of consultation, 747
veterinary, 91
Etiology of dysentery, 503
European veterinary institutions, 620, 689
Examinations for soundness, 140
Excision of the eyeball, 233
Experiments with mallein; glanders and its suppression, 96
Explosive formaldehyde mixture, 503
Experiment station veterinarian as a member of the State Board of Health, the, 81
Extracts from foreign journals (German):
Agricultural districts, veterinary lectures in, 226
Extracts from foreign journals (German):
Dog, operation for constipation on the intestines and stomach of a, 162
Deut. Thierarztliche Wochenschrift, 359
Falotte, 359
Hoskins, J. Preston, Ph.D., 160, 226, 356
Il veterinario, 359
La Revue des Hongreurs, 359
Lung disease in the German Empire during the year 1896, the spread of, 358
Mares, castration of, by Schwendimann in Bern, 160
Meat, after carbolineum has been taken, carbol odor in, 161
Moon-blindness in army horses, the course and diagnosis of, 226
Mules, trichorrhexis nodosa in, 357
Ophthalmics, history of comparative, 356
Periodicals in foreign lands, new veterinary, 359
Revue Medicale, 359
Tuberculin, attempt to infect the ass with, 226
Thierartliches Centralblatt, 161, 226
Eyeball, excision of the, 233

Fatal action of formaldehyde, 368
Fearful responsibility, 513
Feed-stuffs, concentrated (new publications), 122
Felton, H. B., V.M.D., 322
Fish, P. A., D.Sc., D.V.S., 429
Flexor metatarsi, injuries to the, 221
Flies, to protect cattle from, 586
Flower, E. P., 446
Fluid extract of hyoscyamus, 583
extracts made with acetic acid, 655
Foreign bodies swallowed by cattle, a few notes on the, 407
Formad, Robert, V.M.D , 210
Formaldehyde, fatal action of, 368
to disguise odor of, 106
Formalin as an antiseptic, 710
as a surgical dressing, 567
Fractured jaw, two cases of, 378

Gangrenous grease, or dermatitis gangrenosa, 469
Gastritis, 417
Genesee Valley Veterinary Medical Association, 124. 238
German extracts, 160, 226, 356
Germicide, effective permanganate, 435
Germol, 366
Giffen, W. A., 195
Glanders and its suppression; experiments with mallein, 12, 96
in Minnesota, State control of, 737
Glassbrenner, V.S., 417
Goubeaud, George J., D.V.S., 16
Grafts of living bone, 584
Grand Rapids Veterinary College, 310
Grange, E. A. A., M.D., V.S., 674
Greater New York in 1899, 45
New York's triumph, 657

Harger, S. J. J., V.M.D., 22, 231, 360, 686, 720
Hawley, Dr., 53
Hay, L., V.S., 132, 731
"Headache powders," deadly, 586
Health, veterinary legislation and State boards of, 237
Heck, W. A., 248
Helmer, Jacob, D.V.S., 747
Hen, the best, 260
Hess, Clyde S., 486
Higgins, Charles H., B.S., D.V.S., 567, 605
History of comparative ophthalmics, 356
Hog-cholera, practicability of the serum-therapy in the treatment of, 151
serum as a remedy for, 342
Hollingsworth, C. E., V.S., 295, 379

Holocaine, 301, 369
Hopkins, G. S., D.Sc., 271
Horse, chronic hydrocephalus in a, 38
doctor, 260
"needle" abscess in the, 362
on the treatment of purpura hemorrhagica with argentum colloidale Credé, in the, 289
sciatic neurectomy in the, 231
the course and diagnosis of moon-blindness in the army, 226
the relations of the ligamentum nuchæ to first cervical vertebra in the, 271
Horses, a new method of castrating, 589
new method of employing charcoal in the treatment of acute indigestion in, 16
epizoötic cellulitis among, 511
Hoskins, J. Preston, Ph.D., 160, 226, 356
Hoskins, W. Horace, D.V.S., 258, 390
Huidekoper, Rush S., M.D., 537, 634
Hunt, V. G., V.S., 569
Hygienic marketing of milk-food, 401
Hypertrophy, castration for prostatic, 233
Hypodermic cathartics, 26, 87
eucaine solution, 369
Hyoscyamus, fluid extract of, 583

"I APPROVE of the organization of a veterinary contingent to the army," 133
Incompatible mixture, an, 301
Incompatibility, iodide of potassium and mercurial ointment, 365
Indiana Veterinary College commencement exercises, 305
Indigestion, acute, in horses, a new method of employing charcoal in the treatment of, 16
Influence of the mind on the diseases of the body; or the difference between veterinary and medical science, the, 205
Injections of artificial serum into the veins, the use of rectal injections of salt water in place of, 364
Injuries to the flexor metatarsi, 221
Inversion of the bladder, or prolapsus vesicæ, in the mare, following parturition, 164
Ileum, perforation of the, 379
Illinois, to the members of the profession in, 42
veterinary legislation in, 372
Medical and Surgical Association, 192
Immunity, an experiment in, 716
Importance of clinical diagnosis, 36
of keeping a case-book and reporting cases, 547
Infectiousness of milk from tuberculous cows, a contribution to the study of, 572
Injection of barium chloride, destruction of jugular vein and carotid artery following, 512
Injury, peculiar and interesting, 446
Inter-State Association of Livestock Sanitary Boards, 799
Inversion of uterus and vagina, 448
Inoculation against Texas fever, 705
Iodine in purpura hemorrhagica, 104
preparation of tincture of, 584
vehicle for, 105
Iodoform, creolin as a deodorant of, 433
Iodoformagen, 235
Iowa State Veterinary Medical Association, 173
Iron, absorption and excretion of, 432
tincture of chloride of, 585

JAW, two cases of a fractured, 378
Jay, Robert, M.D.V., 437, 507, 587, 717, 783
Jobson, George B., V.S., 217, 388
Journal, 1899–1900, 791
another veterinary, 591
Jugular vein and carotid artery, destruction of, following injection of barium chloride, 512

KAISER's sheep dip, 584
Kaupp, B. F., D.V.S., 339
Kelly, Jas. S., sec., 530, 730
Kempner, Walter, 572
Kesler, G. S., V.S., 491
Keystone Veterinary Medical Association, 186
Knight, Emil, V.M.D., 125
Kooker, W. S., V.S., 258, 461

LACOCK, J. STEWART, 258
Law's sheep dip, 583
Lactate of silver, 168
Laminitis, arecoline in, 104
Larynx, spasm of, 295
Leclainche, M. E., 494
Lee, Daniel D., M.D.V., 378
Legislation, report of committee on, 388
Lellman, Wilfred, V.M.D., 38
Leukæmia, 339
Lewis, H. S., D.V.S., 398
Liniment, anodyne, prescription for, 105
Linseed oil, "boiled," 300
List, E. J., V.S., 164
Lockwood, S., V.S., 126, 315, 733
Longacre, E. D., D.V.S., 123
Lupins as plants poisonous to stock, 766
Lung-disease in the German empire during the year 1896, the spread of, 358
Lung-plague, organism of, 8
Lushington, A. M., V.M.D., 333
Lyman, Richard P., M.D.V., 552

MAGNESIUM hydrate as an antidote in poisoning by arsenic, 434
Maine Veterinary Medical Association, 193, 528
Malarial plasmodium, 159
Mallein, experiments with; glanders and its suppression, 12, 96
Manitoba Veterinary Association, 196
Manley, Dr., 511
Mare, complete eversion of uterus in a, 378
inversion of the bladder, or prolapsus vesicæ, following parturition in the, 164
Mares, castration of, by Schwendimann in Bern, 160
Marriages, 120, 172, 516, 658, 793
Marshall, C. J., V.M.D., 346
Martin, W. J., M.D.C., 42, 104, 167, 235, 300, 364, 432, 501, 655, 710
Massachusetts Veterinary Association, 398
Mastitis, prescription for, 106
McCurdy, F. C., V.S., 560
McFarland, Joseph, M.D., 639, 694
McGill University, veterinary department of, 48, 307
McGregor, James, V.S., 51, 129, 194, 397
McKenzie, J. C., V.S., 205
McKillip Veterinary College (commencement exercises), 306
McMackin, W. C., 126
McNeal, J. H., V.M.D., 231
Meat, carbol odor in, after carbolineum has been taken, 161
inspection, 742
and milk inspection, 477
in small cities and towns, 671
service, suggestions made by a committee of veterinarians to the Mayor of Philadelphia relative to, 646
Meats, toxic ptomaïns of preserved, 169
Median neurectomy, 273
Medicaments which age affects, 582
Medicinal value of sarsaparilla, 366
Medicine, American veterinary, 634
Megowan, C. L., V.S., 52
Menthyl-violet, 586
Merillat, L. A., V.S., 36, 107, 112, 181, 297, 299, 437, 507, 534, 587, 717, 783, 786
Metatarsi, injuries to the flexor, 221
Method of employing charcoal in the treatment of acute indigestion in horses, 16
Methylene-blue, 368

Michener, J. C., V S., 427
Michigan State Veterinary Medical Association, 195
Veterinary act, 304
Microörganisms, biology of pathogenic, 612
Milk as a culture medium, 713
detection of formaldehyde in, 713
-food and the hygienic marketing of the same, 401
-hygiene, important suggestion in, 793
in Boston, 500
to tell the age of, 712
Millar, John J., D.V.S., 593
Minnesota State Veterinary Medical Association, 130, 730
Missouri Valley Veterinary Association, 241, 528, 730
Mixture, an incompatible, 301
Moon-blindness in army horses, course and diagnosis of, 226
Moore, R. C,, D.V.S., 221, 469
Montana State Board of Veterinary Medical Examiners, 234
Montreal Veterinary Medical Association, the, 50, 127, 194, 395
Muir, E. Stanton, Ph.G., V.M.D., 26, 87
Mules, trichorrhexis nodosa in, 357
Municipal ownership of slaughter-houses, an arrangement for, 199
*versus* State control of tuberculosis, 75
Myoneurectomy for cribbing, 720

NASAL discharges, clinical diagnosis of, 441, 507
Naturalist, the veterinarian as a, 265, 350
Nebraska Veterinary Medical Association, 249
Necrology:
Burns, Edward D., 172
Conrow, A. E., V.M.D., M.D., 374
Dougherty, Mrs. William, 375
Dunham, M. W., 172
Ebner, Charles F., V.S., 47
Ferguson, J. W., V.S., 451
Hodgson, Joseph R., Sr., D.V.S., 374
Jennings, Robert, Jr., 375
Kay, Richard, M.D., D.V.S., 374
Meyer, John C., Sr., V.S., 119
Rayner, Harry B., V.S., 47
Rose, James M., V S., 451
Smith, Henry C., V.S., 120
John J., D.V.S., 725
Treacy, Michael J., M.R.C.V.S., 516
"Needle" abscess in the horse, 362
Neurectomy in the horse, sciatic, 231
median, 273
New England Association of the American Veterinary College, State of Massachusetts, 398
Jersey, Veterinary Medical Association of, 125, 315, 793
publications, 121, 304
Feed-stuffs, concentrated, 122
Hanson, Harry D., D.V.S., 121, 122
Medicine, practice of equine, 121
Pennsylvania, annual report of the State Veterinarian of, 304
Prescription writing, 122
York College of Veterinary Surgeons, commencement exercises of, 305
County, Veterinary Medical Association of, 58, 123, 195, 250, 322, 397
in September, 515
scotch these bills in, 119
story of, briefly told, 659, 728, 796
triumph, 657
Newer silver salts, therapeutics of the, 167
Nightshade, or belladonna, deadly, 491
Niles, W. B , D.V.M., 151
Noack, Otto G., V.S., 277, 462.
Nocard, Prof. Ed., 1
Nolan, L. A., 127, 195, 249
North America, Association of Veterinary Faculties and Examining Boards of, 521
North Carolina Veterinary Medical Association, 126
-west, how they advertise in the, 527
Notes, 35, 41, 44, 47, 49, 66, 101, 117, 120, 122, 154, 162, 258, 303, 331, 355, 459, 524, 526, 554, 708
on foreign bodies swallowed by cattle, 407
Now for army recognition, 45
for New York, September, 1899, 370
for the Senate, 118
Nux vomica in parturient apoplexy, 449

ŒSOPHAGOTOMY, 99
Oil, deodorized petroleum, 365
Ointment, incompatibility of iodide of potassium and mercurial, 365
Omaha, what I saw at, 282
Ontario Veterinary Association, 60
College, 311, 382
Operation of docking for fashion, shall the veterinarian perform the? 43
Operative treatment, cribbing and its, 22
Ophthalmics, history of comparative, 366
Organism of lung-plague, the 8
Osteoporosis, synopsis of a report on, 346
Our Mecca in 1900, 658
profession's loss, 514
Ovariotomy on the bitch, 85

PAIGE, JAMES B., D.V.S., 620, 689
Palmer, H. F., 477
Pancreatic secretion, 583
Paralysis of the bowels, a case of, 102
Parasitic diseases of rabbits, 497
Parker, Joseph W., 91
Parturient apoplexy, eserine and barium chloride in, 445
nux vomica in, 449
Paste, escharotic, 300
Patterson, H. G., D. V. S., 482
Pearson, Leonard, B. S., V. M. D., 8, 42, 199, 258, 709
Peculiar and interesting injury, 446
Pennsylvania, 453
annual report of the State Veterinarian of, 304
as to State Veterinarian of, 42
State meeting, 597
Veterinary Medical Association, 132, 251, 316, 386, 461
Veterinary Department, University of, 458
Pepsin and pancreatin, action of boric acid and borax on, 434
Perforation of the ileum, 379
Personals, 62, 117, 120, 197, 262, 327, 355, 375, 399, 467, 534, 600, 670, 733, 804
Peters, A. T., 250
Petty, J. W., 126
Phenol pastilles, 434
Phenosalyl, 369
Phillips, W. S., V. S., 448
Pigs, santonin for worms in, 367
Pills, creosote, 106
Plasmodium, malarial, 159
Plea for the more general use of anæsthesia in veterinary surgery, 681
Poisoning, alcohol as an antidote for carbolic-acid, 366
treatment of carbolic-acid, 436
Poisonous plants, 432
Porter, E. C., 449
Post-graduate course, stock farm veterinary practice as a, 333
Practicability of the serum-therapy in the treatment of hog cholera, 151
Practice of Equine Medicine (new publications), 121
Preparation of tincture of iodine, 584
Prescription Writing (new publications), 122
Preserved meats, toxic ptomaïns of, 169
Prognosis, 427
Pseudo-scab, 150
Purified coal tar, 712

Purpura hemorrhagica, iodine in, 104
on the treatment of, in the horse with argentum colloidale Credé, 289
Purulent conjunctivitis, 106
Pyoktanin, 586

QUINICHLORAL, 712
Quittor, 717

RABBITS, parasitic disease of, 497
Rabietis bacillus, the, 230
Rabies in cattle, 787
in England, 494
report of a case of, 510
Rabinowitch, Lydia, Dr., 572
Ranck, E. M., V.M.D., 510, 639, 694
Ravenel, Mazyck P., M.D., 1, 163, 494
Rayner, James B., V.S., 102
Rectal injections of salt water in place of injections of artificial serum into the veins, 364
Reinhart, N. E., 413
Relation of the ligamentum nuchæ to the first cervical vertebræ in the horse, the, 271
Relations which exist between human tuberculosis and bovine tuberculosis, on the, 1
Remedy, an effective, 503
Report of Committee on Army Legislation, 598
Reports of cases:
Azoturia in a dog, case of, 376
Barium chloride, destruction of jugular vein and carotid artery following injection of, 512
Black, Judson, V.S., 445
Bowels, a case of paralysis of the, 102
Choking, case of, 448
Dickson, Robert, D.V.S , 376
Dog, case of azoturia in a, 376
Eddy, William, 787
Epizoötic cellulitis among horses, 511
Eserine and barium chloride in parturient apoplexy, 445
Flower, E. P., 446
Harger, S. J. J., V.M.D., 720
Hollingsworth, C. E., V.S., 295, 379
Horse, chronic hydrocephalus in a, 38
Horses, epizoötic cellulitis among, 511
Ileum, perforation of the, 379
Injury, peculiar and interesting, 446
Jaw, two cases of fractured, 378
Larynx, spasm of, 295
Lee, Daniel D., M.D.V., 378
Lellman, Wilfred, V.M.D., 38
List, E. J., V.S., 164
Manley, Dr., 511
Mare, complete eversion of uterus in a, 378
inversion of the bladder, or prolapsus vesicæ, following parturition, in the, 164
Myoneurectomy for cribbing, 720
Parturient apoplexy, eserine and barium chloride in, 445
nux vomica in, 449
Phillip, Walker S., 448
Porter, E. C., 449
Ravenel, Mazyck P., M.D., 163
Rabies, case of, 510
in cattle, 787
Ranck, E. M., V.M.D., 510
Rayner, James B., V.S., 102
Tail, amputation of the, 103
Tuberculin *vs.* physical examination, 297
Tuberculosis in a calf, a case of fœtal, 163
Vagina, inversion of uterus and, 448
Warner, George L., 512
Wattles, J. H., M.D., D.V.S., 103
Willard, S. B., V.S., 297
Repp, John J., V.M.D., 547, 671
Requiescat in pace, 793
Resident State Secretaries of the American Veterinary Medical Association, list of, 60
Reynolds, M. H., D.V.M., 81, 737

Rheumatic liniment, 503
Rhoads, W. L., 192, 463
Ridge, W. H., V.M D., 99, 187, 258
Rose, J. Milton, V.S. (necrology), 451
Rumenotomy, bovine, 223
Ryan, L. D., V.S., 425
Ryder, J. E., D.V.S , 140

SALLEY, I. L., D.V.S., 194, 528
Salmon, D. E., 600
Salt solution, value of, 436
Sanitarians, veterinarians as, 155
Sanitary control, 781
Sanitation, dairy, 473
Santonin for worms in pigs, 367
Sarsaparilla, medicinal value of, 366
Scab in sheep, 585
Schütz, J. W., 8
Schuylkill Valley Veterinary Medical Association, 123
Sciatic neurectomy in the horse, 231
Scotch these bills in New York, 119
Secretion, pancreatic, 583
Selection, 289, 494, 649, 705, 774, 780
"Senate, now for the," 118
September, New York in, 515
Serum as a remedy for hog cholera, 342
-therapy in the treatment of hog cholera, practicability of, 151
Seventh International Congress of Veterinary Surgeons, 325
Sheep dip, Kaiser's, 584
Law's, 582
scab in, 585
Sheldon, A. J., D.V.S., 399
Shoe-boil, surgical treatment of, 232
Silver anniversary of American Veterinary College, 522
salts, therapeutics of the newer, 167
Simple test for the detection of albumin in urine, 429
Slaughter-houses, an arrangement for municipal ownership of, 199
Sloan, J. A., B.S., M D.V., 297, 437, 507, 587, 717, 720, 751, 783
Smith, Vet.-Major, 649
Soap, rubefacient, prescription for, 86
Society notes, 48
Society proceedings:
American Veterinary Medical Association, 60
California State Veterinary Medical Association, 51
Chicago Veterinary Society, 52, 180, 390, 463, 530, 594, 731
Genesee Valley Veterinary Medical Association, 124
Illinois Veterinary Medical and Surgical Association, 192
Iowa State Veterinary Medical Association, 173
Keystone Veterinary Medical Association, 186
Lockwood, S., Sec., 126, 315, 732
Maine Veterinary Medical Association, 193, 528
Manitoba Veterinary Association, 196
Massachusetts Veterinary Association, 398
Michigan State Veterinary Medical Association, 195
Minnesota State Veterinary Medical Association, 130, 730
Missouri Valley Veterinary Association, 241, 528, 730
Montreal Veterinary Medical Association, the, 50, 127, 194, 395
Nebraska Veterinary Medical Association, 249
New England Association of the American Veterinary College, State of Massachusetts, 398
North Carolina Veterinary Medical Association, 126

Society proceednigs;
Ontario Veterinary Association, 60
Pennsylvania State Veterinary Medical Association, 132, 251, 316, 386, 461, 597
Resident State Secretaries of American Veterinary Medical Association, list of, 60
Schuylkill Valley Veterinary Medical Association, 123
Seventh International Congress of Veterinary Surgeons, 325
Tennessee Veterinary Medical Association, 803
Veterinary Medical Association of New York County, 58, 123, 195, 250, 322, 397, 732
Veterinary Medical Association of New Jersey, 125, 315
Veterinary Medical Society of University of Pennsylvania, 126, 194, 248, 802
Veterinary Medical Association of District of Columbia, 129, 324
West Chester County Veterinary Medical Association, 528
Wisconsin Society of Veterinary Graduates, 315
Solution for burns, 364
for conjunctivitis, 106
for eczema, 586
Soundness, 594
examination for, 140
vices and their relation to, 53
Spasm of the larynx, 295
Spavin, 649
Sprague, J. D., V.S., 342
Squibb's compound alum powder, prescription for, 106
State Board of Health, the Experiment Station Veterinarian as a member of the, 81
control of tuberculosis, municipal *vs.* 75
Secretaries, list of Resident, 60
Stewart, S., V.S., M.D., 71, 215
Stiles, Ch. Wardell, Ph.D., 240
Stock-farm veterinary practice as a post-graduate course, 333
Story of New York briefly told, 659, 728, 796
Stringhalt, clinical diagnosis of, 112
Surgery, advantages of antiseptic, 590
Surgical items, 114, 299, 444, 786
Surgical treatment of shoe-boil, 232
Swain, S. H., V.S., 233
Sweetapple, C. H., 61
Swine notes, 240
Synopsis of a report on osteoporosis, 346

TAIL, amputation of the, 103
Tanalbin, 367
Tasker, H. Karslake, M.R.C.V.S., 774
Temperature and its relation to soundness, 464
Tenaline, 583
Test for the detection of albumin in urine, 429
Tetanus, 425
toxin and antitoxin, 639, 694
Texas fever, inoculation against, 705
Therapeutical and pharmaceutical notes:
Absorption and excretion of iron, 432
Acetic acid, fluid extracts made with, 655
Acoin, 365
Action of boric acid and borax on pepsin and pancreatin, 434
Adulteration of drugs, 712
Alcohol as an antidote for carbolic-acid poisoning, 366
Alum powder (Squibb's), prescription for compound, 106
Anæsthetic mixture, E. C. P., 105
Antiseptic, formalin as an, 710
solution, prescription for, 706
Bones, graft of living, 584
Bottles, cork-stoppered bottles, 584
Burns, solution for, 364
Calomel, origin of, 716

Therapeutical and pharmaceutical notes:
Camphor as an antidote to carbolic acid, 586
Cancer, treatment of, 105
Carbolic-acid poisoning, treatment of, 436
Carbol-sapol, 367
Cattle, to protect, from flies, 586
Cerate of cantharides, 585
Chloride of iron, tincture of, 585
Compound tincture of opium, prescription for, 585
Chinosol, 301
Chloroform, testing the purity of, 713
Coal tar, purified, 712
Cocaine hydrochlorate incompatible with chloroform water; new test for cocaine, 365
solution, 369
Conjunctivitis, solution for, 106
purulent, 106
Cough, prescription for, 169
Creolin as a deodorant of iodoform, 433
Creosote, adulteration of, 369
pills, prescription for, 106
to render soluble in water, 433
Deadly headache powders, 586
Decomposition of chloroform, 583
Digitalis leaves, constituents of, 582
Dog soap, 584
Domestic animals, a treatise on surgical therapeutics of, 501
Drugs, adulteration of, 712
what becomes of some spoiled, 235
where some come from, 711
which should not be dispensed in capsule form, 583
Dysentery, etiology of, 503
Eczema, solution for, 586
Formaldehyde, fatal action of, 368
in milk, detection of, 713
mixture, explosive, 503
to disguise odor of, 106
Formalin as an antiseptic, 710
Germicide, effective permanganate, 435
Germol, 366
Holocaine, 301, 369
Hypodermic eucaine solution, 369
Hyoscyamus, fluid extract of, 583
Immunity, experiments in, 716
Injections of artificial serum into the veins, the use of rectal injections of salt water in place of, 364
Iodide potassium, prescription for, 503
Iodine in purpura hemorrhagica, 104
preparation of tincture, 584
vehicle for, 105
Iodoformagen, 235
Kaiser's sheep dip, 584
Laminitis, arecoline in, 104
Law's sheep dip, 583
Liniment, prescription for anodyne, 105
Martin, W. J., M.D.C., 104, 167, 235, 300, 364, 437, 501, 582, 655, 710
Mastitis, prescription for, 106
Meats, toxic ptomaïns of preserved, 167
Medicaments which age affects, 582
Methylene-blue, 368
Milk as a culture medium, 713
to tell the age of, 712
Mixture, an incompatible, 301
Oil, "boiled" linseed, 300
deodorized petroleum, 365
instability of phosphorated, 436
Ointment, incompatibility of iodide of potassium and mercurial, 365
Pancreatic secretion, 583
Paste, escharotic, 300
Pepsin and pancreatin, action of boric acid and borax on, 434
Phenol pastilles, 434
Phenosalyl, 369
Photographs, error in X-ray, 432
Pigs, santonin for worms in, 367

Therapeutical and pharmaceutical notes;
Poisonous plants, 432
Pyoktanin, methyl-violet, 586
Rheumatic liniment, 503
Salt solution, value of injection of, 436
Sarsaparilla, medicinal value of, 366
Sheep, scab in, 585
Tanalbin, 367
Tenaline, 583
Tetanus, carbolic acid in the treatment of, 235
Therapeutics of the newer silver salts, 167
the time element in, 300
Tinctures, incompatibilities of, 436
Tuberculin trials, review of, 713
Tuberculosis, for a more accurate diagnosis of, 434
U. S. Pharmacopœia, doses in the, 713
Vanadin, 367
Thorburn, W. W., 419
Tin boxes, to fasten labels on, 37
Tincture of chloride of iron, 585
of iodine, preparation of, 584
of opium compound, prescription for, 585
Tinctures, incompatibilities of, 436
Toxic ptomaïns of preserved meats, 169
Travis, A., V.S., 193
Treatise on surgical therapeutics of domestic animals, 501
Treatment, cribbing and its operative, 22
of acute indigestion in horses, a new method of employing charcoal in, 16
of cancer, 105
of carbolic-acid poisoning, 436
of hog cholera, practicability of the serum-therapy in, 151
of purpura hemorrhagica in the horse with argentum colloidale Credé, on the, 289
of tetanus, carbolic acid in the, 235
Trephining, its indications, technique, after-treatment, and sequelæ, 297, 437, 587
Trichorrhexis nodosa in mules, 357
Tuberculin, attempt to affect the ass with, 226
trials, review of, 713
*versus* physical examination, 297
Tuberculosis, 569
Congress, the Berlin, 239
for a more accurate diagnosis of, 434
in dairy cattle, and how shall we get rid of it, 413
in a calf, a case of fœtal, 163
municipal *versus* State control of, 75
on the relations which exist between human and bovine, 1
Tumors, clinical diagnosis of, 210
Turner, J. P., V.M.D., 130, 325, 681

UDDER and teats, diseases of the, 488
Underhill, B. M., V.M.D., 44
United States College of Veterinary Surgeons, 306
University of Pennsylvania, Veterinary Department commencement exercises, 458
Veterinary Medical Society of the, 126, 194, 248
Urinary analysis, 217
Urine, a simple test for the detection of albumin in, 429
Use of rectal injections of salt water in place of artificial serum into the veins, the, 364
Uterus and vagina, inversion of, 448

VACCINE, my experience with black-leg, 219
Vagina, inversion of the uterus and, 448
Valedictory address, 486
Vanadin, 367
Vehicle for iodine, 105
Vertebra in the horse, the relation of the ligamentum nuchæ to the first cervical, 271
Veterinarian as a naturalist, the, 265, 350
Veterinary Association of the District of Columbia, 129, 324
college, 591
contingent to the army, I approve of the organization of, 133
Department of McGill University, 48, 307
Department, University of Pennsylvania, commencement exercises, 458
ethics, 91
Hospital, University of Pennsylvania, clinical reports, 231, 360
institutions, European, 620
journal, another, 592
lectures in agricultural districts, 226
legislation and State Boards of Health, 237
in Illinois, 372
Medical Association of New Jersey, 125, 315, 732, 793
of New York County, 58, 123, 195, 250, 322, 397
Society of the University of Pennsylvania, 126, 194, 248
medicine, American, 634
periodicals in foreign lands, 359
practice, antisepsis in, 482
electricity and its use in, 419
stock-farm as a post-graduate course, 333
profession's mecca in 1899, 452
Surgeon Desmond, 497
Surgeons, seventh International Congress of, 325
Veterinarians as sanitarians, 155
Veterinorum, echinococcus, 215
Vices and their relations to soundness, 53

WALKER, DR. ROBERT G., 391, 393
Walker, George L. V.S., 512
Warner, J. B., 414
Washburn, H. J., 612
Water, to render creosote soluble in, 433
Wattles, J. H., M.D., D.V.S., 103
West Chester County Veterinary Medical Association, 528
What I saw in Omaha, 282
Wheeler, Arthur S., 407
Where in 1900? 723
Wilcox, E. V., Ph.D., 115, 766
Willard, S. B., V.S., 297
Wisconsin Society of Veterinary Graduates, 315
Wright, J. B., D.V S., 758
Wright, J. H., M.D.C., 12, 96
Wyman, W. E. A., M.D.V., V.S., 36, 37, 107, 114, 297, 437, 507, 587, 717, 783

YAHLBUSH, H. C., 115

